# Association between continuity of care and long-term mortality in Taiwanese first-ever stroke survivors: An 8-year cohort study

**DOI:** 10.1371/journal.pone.0216495

**Published:** 2019-05-22

**Authors:** Chun-Pai Yang, Hao-Min Cheng, Mei-Chun Lu, Hui-Chu Lang

**Affiliations:** 1 Department of Neurology, Kuang Tien General Hospital, Taichung, Taiwan; 2 Department of Medical Research, Kuang Tien General Hospital, Taichung, Taiwan; 3 Department of Nutrition and Institute of Biomedical Nutrition, Hung Kuang University, Taichung, Taiwan; 4 Department of Medical Education, Taipei Veterans General Hospital, Taipei, Taiwan; 5 Division of Cardiology, National Yang-Ming University, Taipei, Taiwan; 6 Department of Public Health, National Yang-Ming University, Taipei, Taiwan; 7 Center for Evidence-based Medicine, Taipei Veterans General Hospital, Taipei, Taiwan; 8 Department of Medical Research, Kuang Tien General Hospital, Taichung, Taiwan; 9 Department of Nuring, Hung Kuang University, Taichung, Taiwan; 10 Institute of Hospital and Health Care Administration, National Yang-Ming University, Taipei, Taiwan; University of Ioannina School of Medicine, GREECE

## Abstract

**Background:**

Continuity of care is considered to be an important principle of stroke care; however, few analyses of empirically related outcomes have been reported.

**Objective:**

This study examined the correlation between the continuity of care for outpatients after a stroke event and the survival of stroke patients over the year following hospital discharge.

**Research design:**

Data from the Taiwan National Health Insurance Database were used in this study. We defined stroke as the ICD-9-CM codes 430 to 437, and all patients were followed up regarding their survival for at least one year. The modified modified continuity index (MMCI) was used as the indicator of continuity of care. Cox proportional hazard models with robust sandwich variance estimates were employed to analyze the correlation between continuity of care and stroke-related death.

**Results:**

A total of 9,252 stroke patients were included in the analysis. Those patients who had a high and a completed COC had a higher percentage of survival (97.25% and 95.39%) compared to the other two groups. After controlling for other variables, compared with the low-level continuity of care group, the moderate-level, high-level and completed continuity of care groups still showed a significantly lower risk of death HR (95% CI) were: 0.63 (0.49–0.80), 0.56 (0.40–0.79) and 0.50 (0.39–0.63), respectively.

**Conclusion:**

Continuity of care may increase the survival among stroke patients and therefore plays an important role in management of stroke after survival.

## Introduction

Continuity of care refers to the establishment of long-term and sustained interactions between a patient and a care provider that involve information continuity, management continuity and relationship continuity. Information continuity refers to the effective sharing of the patient’s medical treatment information by various hospitals or care providers. Management continuity refers to the provision of continuous and integrated care management. Relationship continuity refers to the establishment of a sustained patient-caregiver relationship.[1–5]

Non-quantitative indicators of continuity of care can be divided into subjective and objective indicators.[6–8] Quantitative indicators can be categorized into three types: the density type (measured by the usual provider continuity (UPC) index),[9] the dispersion type (measured by the continuity of care index (COCI)[10] and the modified modified continuity index (MMCI))[11] and the sequential type (measured by the sequential continuity (SECON) index).[12] Compared with residents in other countries, the hospital visit frequency of the Taiwanese population is higher, and this difference may be due to the no compulsive referral system of Taiwan health insurance in Taiwan. Therefore, it is more pertinent to use the dispersion-type indicators, such as the COCI and MMCI, to eliminate the interference that a high medical treatment frequency might have on the measurement accuracy of the indicators.

Stroke is the third most common cause of death in Taiwan and has been an important medical, long-term care and public health problem in this country.[13, 14] Continuity of care for stroke patients includes the prevention and controlling of risk factors before disease onset, the identification of the disease’s symptoms, appropriate and timely medical treatment, the admittance of the patient to a hospital to receive prompt treatment, and the continuity of care after patient discharge. It has been reported that approximately 51% of stroke patients will suffer from a stroke again or will die within one year, with an average one-year mortality rate of 18%. This finding indicates that the long-term continuity of care for stroke patients after discharge is very important. It has also been found that after discharge, stroke patients who received post-acute integration care have lower mortality rates, but investigations into the continuity of outpatient care for discharged stroke patients and its relationship with prognosis have rarely been reported.[15, 16] This study aims to examine the association between the continuity of outpatient care for discharged stroke patients over the one year after discharge and their survival.

## Materials and methods

### Data sources and sample selection

This study used a population-based longitudinal dataset, which consisted of 1 million randomly selected individuals from the year 2005 representative population of insured persons living in Taiwan. The original identification number was encrypted to protect privacy, and this was consistently applied to all such numbers. In this way, it was feasible to follow up the condition of patients by linking every claim belonging to the same patient within the National Health Insurance Research Database (NHIRD) dataset (application and agreement number NHIRD-104-240). The accuracy of the data and diagnoses retrieved from the database has been validated.[17] The study protocol was reviewed and approved by the Institutional Review Board of National Yang-Ming University, Taiwan (approval no. YM103044E).

#### Inclusion and exclusion criteria

We defined our stroke cases by the ICD-9-CM codes. This classification was done via the primary and secondary diagnoses; specifically, either the presence of codes 430–434 and 436–437 in the profile of the outpatient prescription and treatment dataset or the presence of codes 430–434 and 436–437 as the main discharge diagnosis. Furthermore, the sample individuals were limited to those who were hospitalized for their first stroke and patients received CT/MRI within 30 days. In addition, the data retrieval was limited to discharges during the period between January 1, 2005 and December 31, 2012. All information was initially retrieved from the 2005 Longitudinal Health Insurance Database (LHID).

**Fig 1:**
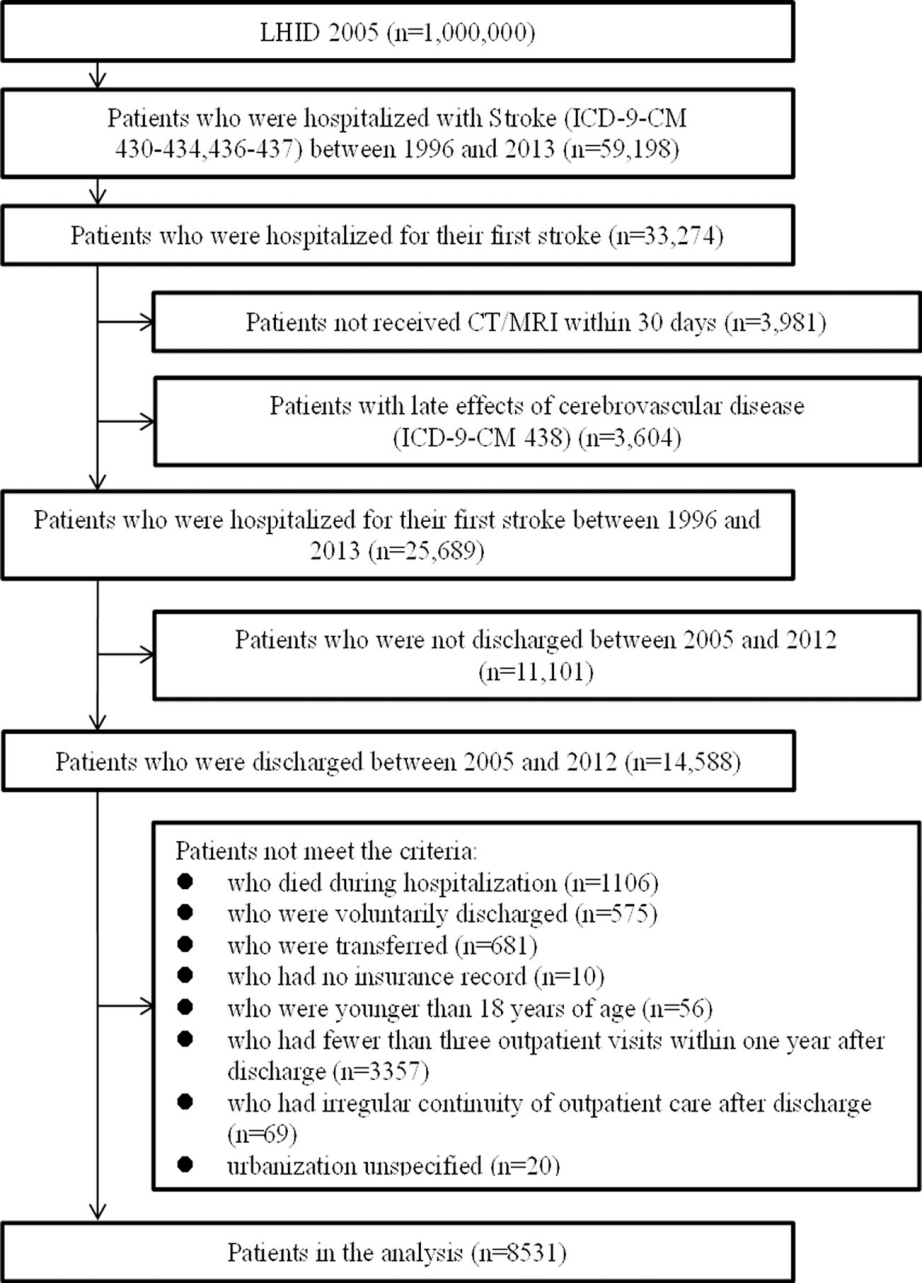
Flow chart of data processing.

We excluded patients who had a past history of stroke (1996–2005), those who were younger than 18 years of age, those who died during hospitalization, those who were voluntarily discharged, those who were transferred, those who had fewer than three outpatient visits within one year after discharge, and those who had irregular continuity of outpatient care after discharge, such as the patient visiting a different physician each time within one year of discharge **(Fig 1)**. The index date was set as the earliest record date for patients who had >1 record during the above period. To avoid time-dependent bias, we examined the COC and the health outcomes of the subjects independently.

### Study variables

#### Independent variables

Regarding the main independent variable, COC, the dispersion-type indicator MMCI was employed for the analysis. MMCI varies between 0 and 1 (with closer to 1 indicating a better continuity of care). We simultaneously considered the total number of medical treatments of the patient and the number of different medical care providers the patients had visited. The MMCI was calculated by entering the number of outpatient visits and the number of medical care providers the patient visited within the first year after discharge into the formula below. Based on these calculations, four groups, namely, low-level, moderate-level, high-level and completed continuity of care, were generated in our analysis.

$$\text{MMCI}=\frac{1-\frac{k}{N+0.1}}{1-\frac{1}{N+0.1}}$$

where k = number of providers and N = total number of visits to all providers.

#### Dependent variables

In this study, the dependent variable was stroke-related death after discharge, and death was defined as in Section 1. In particular, patients were deemed deceased at the hospital if the hospitalization expenses detail profile (DD column) was labeled deceased, and the date of death was the discharge date. Discharged patients were deemed deceased if their insurance was canceled during the follow-up period, if there were no other follow-up medical records and if the date of death was the date of insurance cancelation. The follow-up period was at least one year, until December 31, 2013.

#### Covariates

The covariates included in the analysis were age, gender, premium-based monthly salary, Charlson comorbidity index (CCI),[18]stroke severity index (SSI),[19] tissue plasminogen activator (tPA), outpatient visits, number of deaths, level of hospital, level of hospital doctors were working with, and urbanization level of residency location. Patient age was captured as the age of first hospitalization due to stroke. The patient’s premium-based monthly salary was divided into three categories (≤19,047, 19,048–21,900, and ≥21,901 in New Taiwan Dollars (NTD).

We used the CCI to define the comorbidity of patients. The CCI was calculated based on the data from hospitalizations during the one year before the stroke together with current hospitalization information. It was divided into four groups: 0, 1–3, 4–6, and ≥7 points. The SSI indicator was developed using routine administrative claims data and reflected the stroke severity of patients hospitalized for acute ischemic stroke (AIS). The SSI contains seven predictors: airway suctioning, bacterial sensitivity test, general ward stays, ICU stay, nasogastric intubation, osmotherapy, and urinary catheterization. The SSI has been demonstrated to be a valid proxy for the NIH stroke scale/score (NIHSS) and has been shown to be an effective adjustment for stroke severity in studies of AIS[20] and intracerebral hemorrhage (ICH)[21] outcomes when using administrative claims data. We divided the severity into three levels, namely, low (0–5), moderate (6–12), and high (≥ 13). The outpatient visits within one year after discharge were divided evenly into three groups: low, moderate, and high, according to a count of visits that resulted in primary and secondary diagnoses using ICD-9-CM codes (i.e., diagnosis codes 430–434 and 436 to 437). The number of deaths within one year after discharge was also counted. The level of each hospital was categorized based on the accreditation level of each facility and was divided into medical center, regional hospital, district hospital and nonmedical center. The level of urbanization was determined using the standard established by the National Health Insurance Research Institute of Taiwan. In total, 368 towns in Taiwan have been classified into seven levels, with level 1 representing the most urbanized areas and level 7 representing the least urbanized areas.[22]

### Statistics

The chi-squared test was used to compare the distributions of the basic demographic variables within the three groups that had different levels of continuity of care. Because the information was composed of clustered data, that is, the patient may repeatedly visit the same doctor, in the multivariate analysis Cox proportional hazards models with robust sandwich variance estimates were used to analyze the correlation between continuity of care and stroke-related death. All statistical tests were two-tailed and were conducted using SAS 9.4 (SAS Institute Inc., Cary, North Carolina, USA). A P value of <0.05 was defined as being significant.

## Results

A total of 14 hospitalized patients with a primary diagnosis of stroke and a discharge date during the period from 2005 to 2012were included. Those who had a past history of stroke, those who died during hospitalization, those who were voluntarily discharged, those who were voluntarily discharged and transferred, and those who were younger than 18 years of age were excluded. To avoid bias when calculating the indicators of continuity of care when the frequency of medical treatment was too low, those who visited the hospital fewer than three times within one year after discharge and those who had complete noncontinuity of caregivers were also excluded. Ultimately, a total of 8,531 patients were included in the analysis (Fig 1).

Table 1 shows the basic demographic characteristics of the stroke patients and their distribution among the continuity of care levels. There is no difference in COC between male and female subjects. Compared to other higher COC groups, the low-level COC group shows a higher proportion of patients at an older age (≧80 years old: 21.76% vs. 16.81%~19.81%), a higher proportion of patients with a low income (≦NT$19,047: 36.10% vs. 30.78%~33.49%), and a higher proportion of patients with higher disease severity (CCI 4–6: 4.43% vs. 2.75%~3.58%). Most patients had 1–3 comorbidities, and this group accounted for 50.35% of all patients. In the completed-COC category, patients formed a higher percentage of CCI = 0 and CCI≧7. Up to 56.85% of patients were in the mild SSI category, 22.72% were in the moderate SSI category, and 20.43% were in severe SSI categories. Patients who had a completed COC were also found to form a higher percentage of a mild severity of stroke (SSI≦5), but were found to form a lower percentage of a higher severity of stroke (SSI≧13). The causal relationships that link CCI and SSI with COC are unclear. Those who were categorized into the low frequency of outpatient visits group (≦7 visits) had a higher percentage of patients (54.59%) with a completed COC. Those patients who had a high and a completed COC had a higher percentage of survival (97.25% and 95.39%) compared to the other two groups.

**Table 1:** Demographic characteristic distribution across the different continuity of care groups.

Variable	Total		COC^a^								*p*-value
			Low		Moderate		High		Completed		
**Number**	N = 8531	%	N = 2100	%	N = 2075	%	N = 1929	%	N = 2427	%	
**Sex**											
** Male**	5054	59.24%	1250	59.52%	1257	60.58%	1104	57.23%	1443	59.46%	0.181
** Female**	3477	40.76%	850	40.48%	818	39.42%	825	42.77%	984	40.54%	
**Stroke age**											
** 19–44**	453	5.31%	114	5.43%	115	5.54%	85	4.41%	139	5.73%	0.001
** 45–64**	3048	35.73%	696	33.14%	736	35.47%	700	36.29%	916	37.74%	
** 65–69**	1054	12.35%	251	11.95%	245	11.81%	267	13.84%	291	11.99%	
** 70–79**	2355	27.61%	582	27.71%	568	27.37%	532	27.58%	673	27.73%	
** 80+**	1621	19.00%	457	21.76%	411	19.81%	345	17.88%	408	16.81%	
**Premium-based monthly salary**											
** ≦NT$19,047**	2836	33.24%	758	36.10%	685	33.01%	646	33.49%	747	30.78%	0.018
** NT$19,048-NT$21,900**	3228	37.84%	749	35.67%	800	38.55%	725	37.58%	954	39.31%	
** ≧NT$21,901**	2467	28.92%	593	28.24%	590	28.43%	558	28.93%	726	29.91%	
**CCI**											
** 0**	3856	45.20%	933	44.43%	943	45.45%	802	41.58%	1178	48.54%	<0.001
** 1–3**	4295	50.35%	1055	50.24%	1057	50.94%	1040	53.91%	1143	47.10%	
** 4–6**	293	3.43%	93	4.43%	57	2.75%	69	3.58%	74	3.05%	
** ≧7**	87	1.02%	19	0.90%	18	0.87%	18	0.93%	32	1.32%	
**SSI**^**b**^											
** Mild**	4850	56.85%	1066	50.76%	1112	53.59%	993	51.48%	1679	69.18%	<0.001
** Moderate**	1938	22.72%	524	24.95%	466	22.46%	478	24.78%	470	19.37%	
** Severe**	1743	20.43%	510	24.29%	497	23.95%	458	23.74%	278	11.45%	
**Outpatient visits**^**c**^											
** Low**	2707	31.73%	780	37.14%	602	29.01%	0	0.00%	1325	54.59%	<0.001
** Moderate**	2926	34.30%	824	39.24%	529	25.49%	694	35.98%	879	36.22%	
** High**	2898	33.97%	496	23.62%	944	45.49%	1235	64.02%	223	9.19%	
**Number of Deaths**											
** Alive**	8060	94.48%	1898	90.38%	1971	94.99%	1876	97.25%	2315	95.39%	<0.001
** Dead**	471	5.52%	202	9.62%	104	5.01%	53	2.75%	112	4.61%	
**Urbanization**											
** 1**	2669	31.29%	627	29.86%	647	31.18%	617	31.99%	778	32.06%	0.513
** 2**	3834	44.94%	948	45.14%	938	45.20%	847	43.91%	1101	45.36%	
** 3**	581	6.81%	144	6.86%	137	6.60%	137	7.10%	163	6.72%	
** 4**	1288	15.10%	335	15.95%	312	15.04%	287	14.88%	354	14.59%	
** 5–7**	159	1.86%	46	2.19%	41	1.98%	41	2.13%	31	1.28%	
**Hospital Accreditation Level**											
** Medical Center**	3258	38.19%	818	38.95%	846	40.77%	658	34.11%	936	38.57%	<0.001
** Regional Hospital**	3467	40.64%	863	41.10%	810	39.04%	804	41.68%	990	40.79%	
** District hospital**	1006	11.79%	250	11.90%	217	10.46%	266	13.79%	273	11.25%	
** Others**	800	9.38%	169	8.05%	202	9.73%	201	10.42%	228	9.39%	
**Doctor’s Affiliation to Hospital Level**											
** Medical Center**	2564	30.06%	638	30.38%	636	30.65%	389	20.17%	901	37.12%	<0.001
** Regional Hospital**	3018	35.38%	730	34.76%	682	32.87%	644	33.39%	962	39.64%	
** Local Hospital**	1472	17.25%	396	18.86%	361	17.40%	416	21.57%	299	12.32%	
** Clinics**	664	7.78%	164	7.81%	177	8.53%	286	14.83%	37	1.52%	
** Others**	813	9.53%	172	8.19%	219	10.55%	194	10.06%	228	9.39%	

COC, continuity of care; MMCI, the modified modified continuity index; CCI, Charlson comorbidity index; SSI, stroke severity.

^a^ COC was classified as low (MMCI≦0.75), moderate (MMCI: 0.75–0.86), high (MMCI: 0.86–0.99) and completed (MMCI = 1);

^b^ SSI was classified as mild (≦5), moderate (6–12) and severe (≧13);

^c^ Outpatient visits was classified as low (≦7), moderate (8–13) and high (≧14).

The highest proportion (40.64%) of stroke patients was admitted to regional hospitals; this was followed by medical centers (38.19%), district hospitals (11.79%), and others units (9.38%). The levels of hospitals with which the outpatient physician was affiliated before and after discharge showed the same pattern as admission, and regional hospitals had the highest percentage of patient visits. In terms of COC, outpatients with a completed COC had a higher percentage of visiting a medical center and/or a regional hospital. By contrast, outpatients with a completed COC had a lower percentage of visited a local Hospital and/or clinics compared to the other three COC groups.

Table 2 shows the survival analyses of four stroke patient groups with completed, high, moderate, and low levels of outpatient continuity of care. This was created using various Cox proportional hazard models with robust sandwich variance estimates. Without controlling for the other variables, compared with the low-level continuity of care group, the groups with moderate, high and completed levels of continuity showed a significantly lower risk of death (HR = 0.50, 0.27 and 0.47, respectively). After controlling for the other variables, compared with the low-level continuity of care group, the groups with moderate, high and completed levels of continuity showed a significantly lower risk of death HR (95% CI) were: 0.63 (0.49–0.80), 0.56 (0.40–0.79) and 0.50 (0.39–0.63), respectively.

**Table 2:** Overall survival analysis using univariate and multivariate cox regression analysis.

Variable	Crude HR	(95% CI)	*p*-value	Adjusted HR	(95% CI)	*p*-value
**COC**^**a**^						
** Low**	1			1		
** Moderate**	0.5	(0.40–0.64)	<0.001	0.63	(0.49–0.80)	<0.001
** High**	0.27	(0.20–0.37)	<0.001	0.56	(0.40–0.79)	0.001
** Completed**	0.47	(0.37–0.59)	<0.001	0.5	(0.39–0.63)	<0.001
**Sex**						
** Male**	0.94	(0.78–1.12)	0.476	1.2	(0.99–1.44)	0.057
** Female**	1			1		
**Stroke age**	1.07	(1.06–1.08)	<0.001	1.06	(1.05–1.06)	<0.001
**Premium-based monthly salary**						
** ≦NT$19,047**	1			1		
** NT$19,048-NT$21,900**	1	(0.82–1.23)	0.987	1.06	(0.86–1.31)	0.595
** ≧NT$21,901**	0.68	(0.54–0.87)	0.002	0.86	(0.67–1.10)	0.217
**Doctor’s affiliation to Hospital Level**						
** Medical Center**	1			1		
** Regional Hospital**	1.53	(1.19–1.95)	0.001	1.43	(1.10–1.85)	0.007
** Local Hospital**	2.26	(1.74–2.94)	<0.001	1.93	(1.46–2.56)	<0.001
** Clinics**	1.06	(0.69–1.62)	0.791	1.46	(0.94–2.26)	0.091
** Other**	1.41	(0.99–2.01)	0.058	1.25	(0.86–1.82)	0.251
**Urbanization**						
** 1**	1			1		
** 2**	1.14	(0.91–1.41)	0.25	1.02	(0.82–1.28)	0.845
** 3**	1.38	(0.97–1.98)	0.076	1.16	(0.79–1.69)	0.444
** 4**	1.28	(0.97–1.70)	0.079	0.96	(0.71–1.30)	0.797
** 5–7**	1.3	(0.69–2.48)	0.419	0.83	(0.43–1.60)	0.573
**CCI**						
** 0**	1			1		
** 1–3**	1.58	(1.29–1.93)	<0.001	1.41	(1.15–1.73)	0.001
** 4–6**	2.95	(2.01–4.32)	<0.001	2.5	(1.70–3.66)	<0.001
** ≧7**	12.59	(8.67–18.27)	<0.001	9	(6.14–13.19)	<0.001
**SSI** ^**b**^						
** Mild**	1			1		
** Moderate**	1.65	(1.29–2.11)	<0.001	1.59	(1.24–2.03)	<0.001
** Severe**	3.6	(2.93–4.43)	<0.001	3.46	(2.80–4.29)	<0.001
**Outpatient visits**^**c**^						
** Low**	1			1		
** Moderate**	0.39	(0.32–0.49)	<0.001	0.38	(0.30–0.47)	<0.001
** High**	0.28	(0.22–0.36)	<0.001	0.22	(0.17–0.30)	<0.001

HR, hazard ratio; CI, confidence interval; COC, continuity of care; MMCI, the modified modified continuity index; CCI, Charlson comorbidity index; SSI, stroke severity.

^a^ COC was classified as low (MMCI≦0.75), moderate (MMCI: 0.75–0.86), high (MMCI: 0.86–0.99) and completed (MMCI = 1);

^b^ SSI was classified as mild (≦5), moderate (6–12) and severe (≧13);

^c^ Outpatient visits was classified as low (≦7), moderate (8–13) and high (≧14).

Table 3 shows the separate analysis for all the stroke patients into three groups: acute ischemic stroke (AIS), intracerebral hemorrhage (ICH), and spontaneous subarachnoid hemorrhage (SAH). The results were similar to that in Table 2. While some variables became not significant in SAH group, those were still significant in AIS and ICH groups. This variation could be due to the small sample size of SAH group. The results showed that COC is both valid for AIS and ICH. However, COC is not significant in terms of mortality for SAH patients. The tPA therapy was only applied to acute ischemic stroke (AIS) and the results showed no significant effect on the survival.

**Table 3:** Overall survival analysis using univariate and multivariate cox regression analysis.

Variable	Ischemic stroke (n = 6,437)			ICH (n = 1,525)			SAH (n = 165)		
	Adjusted HR	(95% CI)	*p*-value	Adjusted HR	(95% CI)	*p*-value	Adjusted HR	(95% CI)	*p*-value
**COC**^**a**^									
** Low**	1			1			1		
** Medium**	0.63	(0.48–0.83)	<0.001	0.56	(0.30–1.05)	0.07	0.48	(0.03–8.33)	0.62
** High**	0.65	(0.45–0.95)	0.02	0.38	(0.15–0.92)	0.03	0.46	(0.02–14.00)	0.66
** Completed**	0.51	(0.39–0.67)	<0.001	0.44	(0.22–0.85)	0.02	0.71	(0.06–8.62)	0.78
**Sex**									
** Male**	1.2	(0.97–1.47)	0.09	1.05	(0.64–1.72)	0.83	1.39	(0.23–8.50)	0.73
** Female**	1			1			1		
**Stroke age**	1.06	(1.05–1.07)	<0.001	1.05	(1.03–1.06)	<0.001	1.1	(1.02–1.19)	0.01
**Premium-based monthly salary**									
** ≦NT$19,047**	1			1			1		
** NT$19,048-NT$21,900**	1.01	(0.80–1.28)	0.95	1.24	(0.74–2.10)	0.41	0.64	(0.04–11.27)	0.76
** ≧NT$21,901**	0.87	(0.65–1.14)	0.3	0.64	(0.33–1.24)	0.18	3.36	(0.28–39.80)	0.34
**Doctor’s affiliation to Hospital Level**									
** Medical Center**	1			1			1		
** Regional Hospital**	1.52	(1.13–2.05)	0.01	1.01	(0.54–1.89)	0.97	1.98	(0.06–68.81)	0.71
** Local Hospital**	2.02	(1.46–2.80)	<0.001	1.33	(0.64–2.76)	0.45	8.57	(0.50–147.07)	0.14
** Clinics**	1.47	(0.89–2.44)	0.14	1.1	(0.39–3.12)	0.86			
** Other**	1.47	(0.98–2.22)	0.06	0.28	(0.06–1.24)	0.09			
**Urbanization**									
** 1**	1			1			1		
** 2**	1.02	(0.79–1.32)	0.87	1.06	(0.61–1.86)	0.84	0.65	(0.07–6.00)	0.71
** 3**	1.04	(0.66–1.62)	0.87	1.86	(0.77–4.46)	0.17	0.32	(0.01–9.34)	0.5
** 4**	1.09	(0.78–1.53)	0.61	0.73	(0.33–1.61)	0.44			
** 5–7**	1.04	(0.53–2.04)	0.9				-	-	-
**CCI**									
** 0**	1			1			1		
** 1–3**	1.33	(1.05–1.68)	0.02	1.71	(1.04–2.80)	0.03	1.21	(0.14–10.81)	0.87
** 4–6**	2.43	(1.60–3.69)	<0.001	1.74	(0.41–7.43)	0.46	-	-	-
** ≧7**	8.01	(5.26–12.18)	<0.001	18.54	(5.12–67.12)	<0.001	-	-	-
**SSI**^**b**^									
** Mild**	1			1			1		
** Moderate**	1.60	(1.21–2.12)	0.001	1.38	(0.57–3.30)	0.47			
** Severe**	3.83	(3.00–4.90)	<0.001	2.74	(1.20–6.24)	0.02	9.94	(0.67–147.25)	0.09
**Outpatient visits**^**c**^									
** Low**	1			1			1		
** Moderate**	0.34	(0.26–0.44)	<0.001	0.5	(0.28–0.88)	0.02	0.09	(0.01–1.28)	0.07
** High**	0.22	(0.16–0.31)	<0.001	0.21	(0.10–0.44)	<0.001			
**tPA**									
** Yes**	0.90	(0.46–1.76)	0.76						
** No**	1								

ICH, intracerebral hemorrhage; SAH, subarachnoid hemorrhage; HR, hazard ratio; CI, confidence interval; COC, continuity of care; MMCI, the modified modified continuity index; CCI, Charlson comorbidity index; SSI, stroke severity; tPA, tissue plasminogen activator

continuity index; CCI, Charlson comorbidity index; SSI, stroke severity.

^a^ COC was classified as low (MMCI≦0.75), moderate (MMCI: 0.75–0.86), high (MMCI: 0.86–0.99) and completed (MMCI = 1);

^b^ SSI was classified as mild (≦5), moderate (6–12) and severe (≧13);

^c^ Outpatient visits was classified as low (≦7), moderate (8–13) and high (≧14).

## Discussion

Continuity of care is the basis of primary care. The literature shows the benefits of a higher continuity of care for specific diseases. For example, in the case of diabetes, a higher continuity of medication is able to more successfully reduce the readmission rate, emergency room usage, and medical costs.[22–26] In the case of asthma, a higher continuity of outpatient care is able to more effectively decrease next-year emergency room usage in children.[26] Elderly asthmatic patients with a lower continuity of outpatient care have a significantly higher likelihood of having an asthma-related emergency room visit.[27] Some studies have also examined particular patient populations or specific clinical conditions. For example, the relationship between the continuity of care in children, in elderly cancer patients, and in multiple chronic condition patients has been studied in the context of medical usage.[26, 28–32] Our present findings show that the continuity of care for stroke patients is correlated with their survival. This study represents an important empirical research finding based on big data and further emphasizes the significance of continuity of care regarding clinical outcomes.

Previously, the relationship between continuity of care and death had been rarely addressed. Specifically, two long-term follow-up studies have shown that the higher the continuity of outpatient care among the elderly, the lower the risk of death.[33, 34] Several other studies have focused on acute diseases, including heart failure, and found that the higher the continuity of care within one year of discharge, the lower the risk of death within that year.[35] An investigation of the relationship between the continuity of care for patients with cardiovascular diseases and five-year survival revealed that those with a higher level of continuity of care have a lower risk of death.[36] In the present study, the nationally representative data of more than 19,000 stroke patients over a period of eight years has been used to show that continuity of care for patients with cerebrovascular diseases is correlated with their one-year survival, which is similar to the findings for cardiovascular disease in that, after the initial acute care, the continuity of care after discharge was found to be correlated with the patients’ long-term prognoses.

In this study, the MMCI, a dispersion-type indicator, was used to measure continuity of care. The MMCI has been widely used in studies to measure the ratio of a patient’s outpatient visits to a certain physician in relation to the patient’s total outpatient visits during a particular observation period.[6–8, 36] Taiwan implemented a universal health insurance system two decades ago, and as a result, it would appear that the frequency of medical visits in Taiwan is higher than that in a number of other countries. In such a situation, the MMCI is a more suitable measure and should be able to achieve higher measurement accuracy. Continuity of care can also be measured using nonquantitative indicators; these can be categorized into objective and subjective types and will be used in future studies, which will be based on the results of the present quantitative study.

Our results show that compared with other groups, in addition to their poorer survival, the low-level continuity of care group shows a higher proportion of patients at an older age, a higher proportion of patients with a low income, and a higher proportion of patients with higher disease severity. Similar to our results, the results of previous studies have also shown that age, socioeconomic status, and disease severity can affect continuity of care; however, the causal relationship remains unclear and requires further clarification.[27, 37, 38] Ethical and patient clinical care issues mean that it is difficult to design large-scale randomized experiments that will address this causal relationship issue. As a result, in the multivariate analysis, to avoid potential interference we controlled as much as possible for potential interfering factors, including patient-related factors, disease-related factors, age, sex, socioeconomic status, comorbidity level, disease severity, physician-related factors (the level of the hospital with which the physician is affiliated) and medical institution-related factors (the level of the hospital visited by the patient).

This study used a large-scale follow-up cohort dataset with national representativeness, which provides stronger evidence compared to past small-scale studies. The Taiwan National Health Insurance Database has high data accuracy and patient inclusiveness.[24, 30, 39] Moreover, this study presents information regarding all patients with stroke, since stroke as a disease can be categorized into several subtypes, such as intracerebral hemorrhage (ICH), ischemic stroke, subarachnoid hemorrhage (SAH) and others, which accounted for 17.9%, 75.5%, 1.9% and 4.7%, respectively, of the cohort cases (data not shown).

There are limitations that affect this study. First, it lacks information on the lifestyle of patients with cerebrovascular diseases, including smoking, drinking, exercise and other risk factors. Second, the patients’ insurance premiums were used as a proxy to represent the patients’ socioeconomic statuses, without considering education, occupation, or marital status. Third, although past studies have indicated that the CCI can be used to represent comorbidity, this study lacks direct information on these comorbidities, such as diabetes, hypertension, and cancer, as well as the treatments used for those diseases.[40–42] Fourth, while previous studies have indicated that the stroke severity index (SSI) is able to be converted into the NIHSS, in this study we did not directly obtain the NIHSS data.[20, 43] Fifth, our claim datasets lack functional measures. Therefore, we were unable to examine some important clinical and functional covariates with the mortality outcome. This includes examples such as linking the blood pressure reduction, compliance rate, and mRS with survival. A recent investigation has demonstrated that appropriate controls are able to mitigate the effect of the above factors on the findings,[44] and in this study, although controls for interfering factors were applied, care still needs to be taken when extrapolating the findings of this study.
